# Supplementary material for: Development of Bedaquiline-Loaded SNEDDS Using Quality by Design (QbD) Approach to Improve Biopharmaceutical Attributes for the Management of Multidrug-Resistant Tuberculosis (MDR-TB)
Source: Antibiotics (Basel). 2023 Oct 3;12(10):1510. doi: 10.3390/antibiotics12101510 (PMC10603879; doi:10.3390/antibiotics12101510)

Supplementary Tables

Table S1: Responses to selected factors

**Run**	**Factor 1 A: Oil (%)**	**Factor 2 B: S_{mix} (%)**	**Factor 3 C: Sonication time (sec)**	**Response 1 droplet size (nm)**	**Response 2 PDI**	**Response 3 Transmittance (%)**
1	30	50	30	125.9	0.21	93.6
2	10	50	60	94.3	0.168	98.5
3	20	60	60	95.2	0.237	97.8
4	20	50	45	89.8	0.21	96.9
5	20	60	30	83.1	0.19	97.2
6	10	50	30	86.7	0.176	97.8
7	10	60	45	78.4	0.16	98.9
8	30	60	45	119.1	0.236	94.4
9	10	40	45	90.25	0.11	97.3
10	20	40	30	97.5	0.153	95.6
11	30	40	45	130.5	0.325	93.1
12	30	50	60	135.7	0.449	93.9
13	20	40	60	105.6	0.331	96.4
14	20	50	45	88.5	0.2	96.8

Abbreviations: PDI, polydispersity index

Table S2: Quality target product profile (QTPP) postulated for SNEDDS of Bedaquiline

QTPP elements	Target	Justification
**Dosage form**	SNEDDS	Selection of lipid-based solid self-nano emulsifying system helps in the oral bioavailability enhancement of poorly bioavailable drug, Bedaquiline.
**Dosage type**	Sustain release	Faster onset of action leading to enhanced therapeutic benefits.
**Dosage strength**	5 mg	Unit dose of Bedaquiline incorporated in a single formulation of SNEDDS
**Route of administration**	Oral	Recommended route for delivery of Bedaquiline
**Stability**	6 months of Accelerated and long-term stability studies	To maintain therapeutic potential of the drug during storage period
**Container closure system**	Airtight glass bottles	To protect against the degradation of the drug and lipids in the presence of atmospheric air.
**Alternative methods of administration**	Salt, polymorphs, solid dispersions, nano crystals, co-crystals, inclusion complexes	These systems can only improve the dissolution rate (but not the extent) of which may eventually lead only to enhancement in rate of oral adsorption.
**Contraindications**	None	None

Abbreviations: QTPP, quality target product profile; SNEDDS, self-nano emulsifying drug delivery system

Table S3: Critical quality attributes (CQAs) for SNEDDS of Bedaquiline and their justifications.

**CQAs**	**Target**	**Is this a CQA**	**Justification**
Physical attributes colour	Acceptable to patient	No	Color, odor and appearance were not considered as critical, as these are not directly linked to patient efficacy and safety.
Odour and appearance	Acceptable to patient	No	
Assay and content uniformity	100%	No	SNEDDS being the homogenous dispersions containing drug solubilized in the blend of lipidic excipients, these variables were regarded as less critical.
Drug release	100%	Yes	Drug release rate is important for fast absorption of the drug in blood; hence was regarded as highly critical.
Liquefaction time	Low	yes*	Lower value of liquefaction time is important for faster drug release from the dosage form. Thus, it was considered as critical.
Emulsification time	Low	yes*	Lower values of emulsification time help in ease of formation of nanoemulsion; hence was taken up as highly critical.
Globule size	<100nm	yes*	Smaller droplet size allows easy penetration through GI epithelial lining and paracellular pathways; hence was regarded as highly critical.
Mean dissolution time	Low	yes*	It is an indicator of faster and complete drug release solubilization of drug in the dissolution medium, thus taken up as highly critical.
PDI	Low	yes*	Highly important for achieving the therapeutically effectiveness he hence considered as highly critical.

*CQAs considered as critical

Abbreviations: CQAs, critical quality attributes; SNEDDS, self-nano emulsifying drug delivery system, PDI, polydispersity index

Table S4: Protocol of stability study

<table>
<tr><td colspan="3">Name of Product: BDQ-F-SNEDDS</td><td colspan="2">Purpose of Study : New Formulation</td></tr>
<tr><td colspan="3">Batch Size : 100 ml</td><td colspan="2">Batch No. : BDSND22</td></tr>
<tr><td>Sr. No.</td><td>Storage Condition</td><td>Storage Period</td><td>Testing Frequency (Monthly)</td><td>Tests</td></tr>
<tr><td>1.</td><td>40 ± 2 ºC & 75 ± 5 %</td><td>6 Months</td><td>0
3
6</td><td rowspan="2">Following test to be performed at all stations of both Accelerated and Long Term Studies.
Physical Appearance
Phase Separation
Caking
Size (nm)
PDI %
Entrapment Efficiency</td></tr>
<tr><td>2.</td><td>25 ± 2 ºC & 60 ± 5 %</td><td>Upto Shelf Life</td><td>0
3
6
9
12
18
24</td></tr>
</table>

Abbreviations: BDQ-F-SNEDDS, Bedaquiline-fumarate-self-nano emulsifying drug delivery system, PDI, polydispersity index

Supplementary Figures

Figure S1: Pseudoternary phase diagram showing different ratios of S_{mix} 1:0, 1:1, 1:2, 2:1, 3:1, 4:1, 5:1

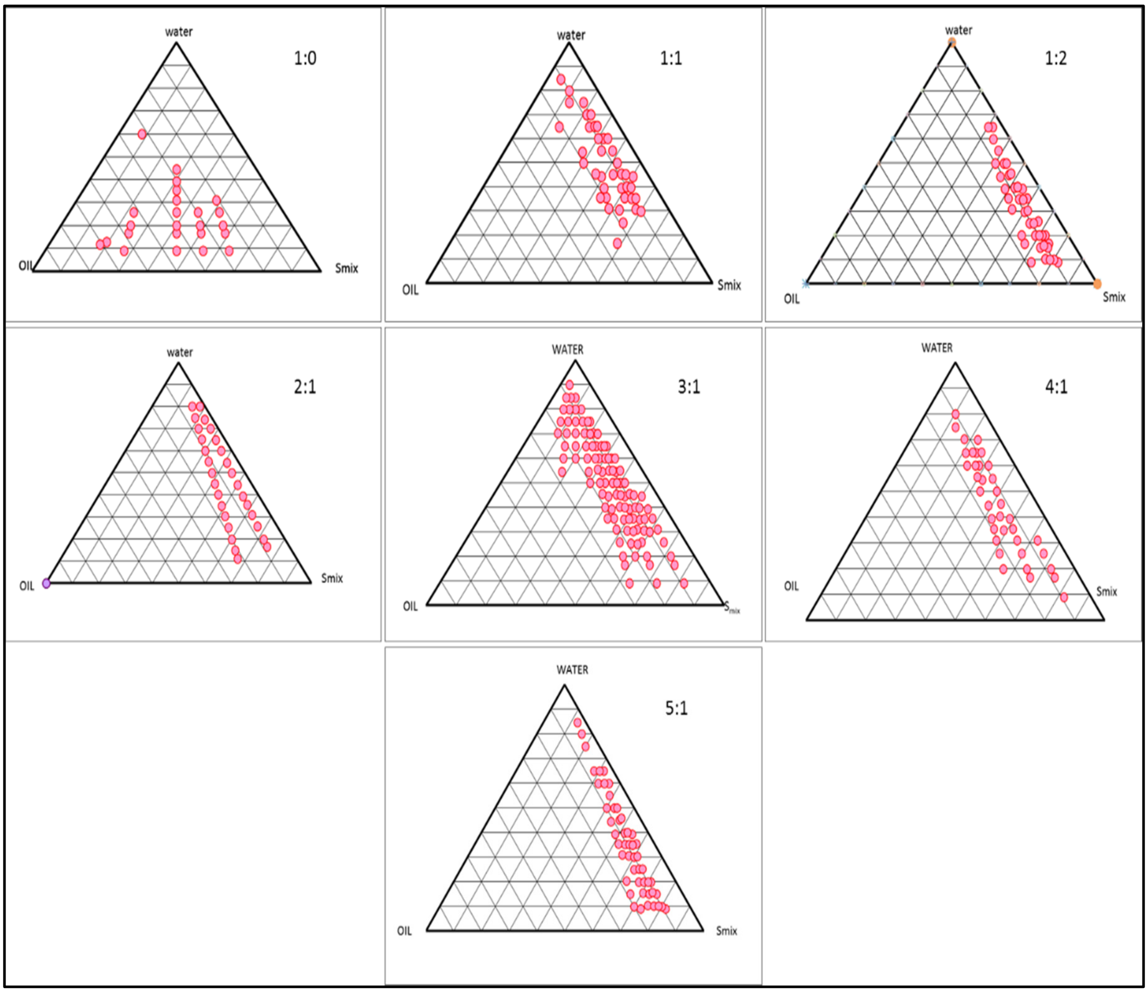

Figure S2: Linear correlation plot between observed and predicted values of droplet size, PDI and Transmittance and their corresponding residual plots.

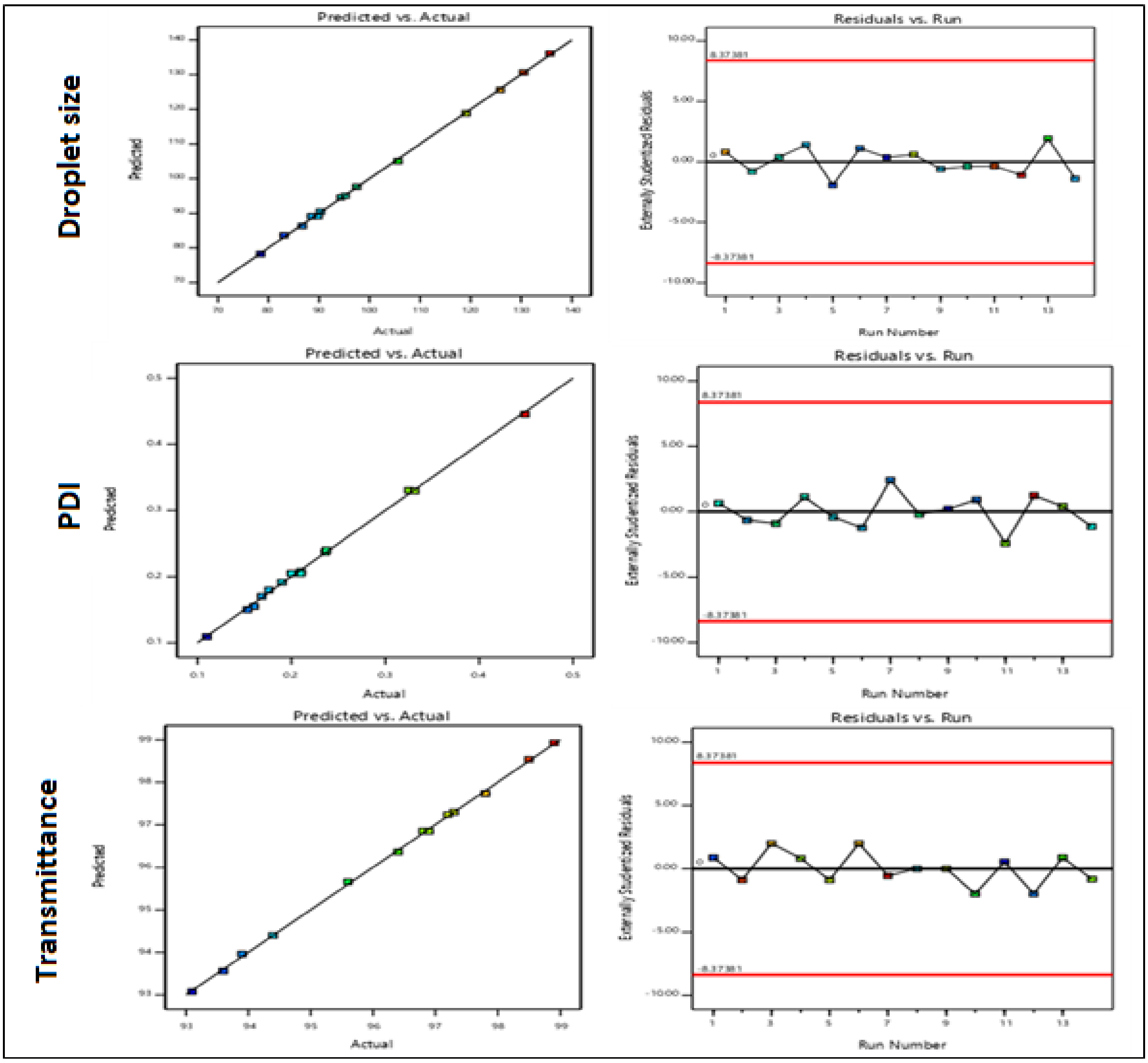

Figure S3: Ishikawa fishbone diagram illustrating a cause-and-effect relationship among the formulation and process variables for the formulation of SNEDDS.

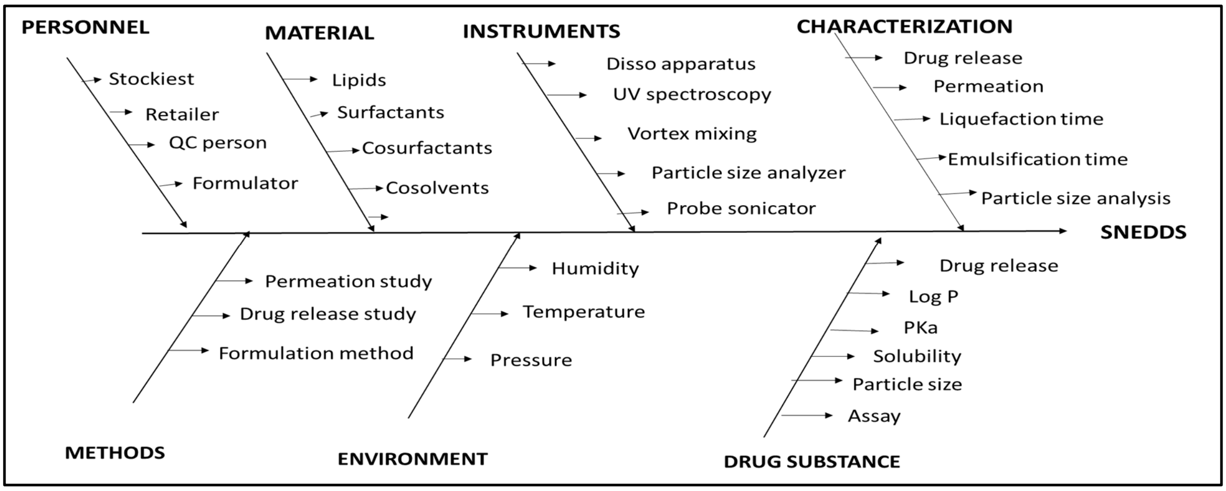

Supplement: Supplementary file 1 [file antibiotics-12-01510-s001.zip › antibiotics-2574122-supplementary.pdf]
